# Extracellular vesicles-mediated intercellular communication: roles in the tumor microenvironment and anti-cancer drug resistance

**DOI:** 10.1186/s12943-019-0965-7

**Published:** 2019-03-30

**Authors:** Selma Maacha, Ajaz A. Bhat, Lizandra Jimenez, Afsheen Raza, Mohammad Haris, Shahab Uddin, Jean-Charles Grivel

**Affiliations:** 1 Division of Translational Medicine, Sidra Medicine, PO BOX 26999, Doha, Qatar; 2 Department of Cell and Developmental Biology, Vanderbilt University School of Medicine, Nashville, TN USA; 3 National Center for Cancer Care and Research, Hamad Medical Corporation, Doha, Qatar; 4 Laboratory Animal Research Center, Qatar University, Doha, Qatar; 5 Translational Research Institute, Academic Health System, Hamad Medical Corporation, Doha, Qatar

**Keywords:** Tumor microenvironment, Stroma, Metastasis, Extracellular vesicles, Drug resistance

## Abstract

The tumor microenvironment represents a complex network, in which tumor cells not only communicate with each other but also with stromal and immune cells. Current research has demonstrated the vital role of the tumor microenvironment in supporting tumor phenotype via a sophisticated system of intercellular communication through direct cell-to-cell contact or by classical paracrine signaling loops of cytokines or growth factors. Recently, extracellular vesicles have emerged as an important mechanism of cellular interchange of bioactive molecules. Extracellular vesicles isolated from tumor and stromal cells have been implicated in various steps of tumor progression, such as proliferation, angiogenesis, metastasis, and drug resistance. Inhibition of extracellular vesicles secretion, and thus of the transfer of oncogenic molecules, holds promise for preventing tumor growth and drug resistance. This review focuses on the role of extracellular vesicles in modulating the tumor microenvironment by addressing different aspects of the bidirectional interactions among tumor and tumor-associated cells. The contribution of extracellular vesicles to drug resistance will also be discussed as well as therapeutic strategies targeting extracellular vesicles production for the treatment of cancer.

## Background

The last decades have revealed that the malignant properties and progression of tumors are not controlled by cancer cells exclusively [1]. The area surrounding the tumor contains various non-malignant cell types, including fibroblasts, lymphocytes, inflammatory cells, endothelial cells, adipose tissue, and mesenchymal stem cells [1]. In the early stages of tumorigenesis, the microenvironment displays anti-tumor immunity and controls tumor growth [2]. As the tumor continues to develop, the role of the microenvironment shifts over to be tumor promotive [2]. Cells found in the tumor microenvironment (TME) have been recognized as key regulators of tumor promotion by providing mitogenic growth factors, growth inhibitory signals or trophic factors [2]. The complex heterotypic interactions between tumor cells and non-cancerous cells within the TME occur through direct contact between cells or paracrine signal exchange of cytokines and growth factors [2]. The most well-recognized cell-to-cell interaction within the TME is between tumor cells and macrophages or fibroblasts [2]. Macrophages play an integral role in host innate immune response against infections [3]. Tumor cells release factors, such as vascular endothelial growth factor (VEGF), colony stimulating factor 1 (CSF1), and platelet-derived growth factor (PDGF), that aid in the recruitment of macrophages to tumors [3]. Once the macrophages are recruited to the tumor, they can promote tumor progression by enhancing tumor cell proliferation, as well as by remodeling the tumor stroma to facilitate invasion and angiogenesis [3]. Fibroblasts are responsible for the production of extracellular matrix (ECM), such as collagen and fibronectin, and facilitate remodeling in wound healing [4]. Cancer-associated fibroblasts (CAFs) support tumor growth, invasion, metastasis and induce inflammation [4]. Stromal cell-derived factor 1 (SDF1) is a CAF-secreted factor that can activate C-X-C chemokine receptor type 4 (CXCR4) and ultimately stimulates cancer cell proliferation [2, 4]. CAF-derived transforming growth factor-beta (TGF-β) promotes the metastatic potential of tumor cells by driving an epithelial-to-mesenchymal transition (EMT) [2, 4].

Recently, it has become apparent that secreted extracellular vesicles (EVs) are proficient intercellular communication mediators [2]. EVs are a heterogeneous population of cell-derived membrane vesicles that are secreted by various cell types. They exhibit a wide size range and differ by their biogenesis. EVs include exosomes, which are small membrane vesicles, ranging from 30 to 150 nm in diameter, and shed microvesicles (MVs), which are large membrane vesicles of 150 to 1000 nm diameter budding off the plasma membrane [5]. Smaller shed MVs have also been reported, which are ~ 100 nm in diameter [6]. Oncosomes are even larger EVs that are also shed off from the plasma membrane and are 1 to 10 μm in diameter [7]. EVs contain a diverse array of bioactive cargoes, including proteins, lipids, and nucleic acid [5, 7, 8]. The lipid bilayer of EVs encapsulates their contents, shielding them from enzymatic degradation [2]. EVs regulate multiple cellular processes including cell proliferation, survival, and transformation through autocrine and paracrine interactions [5, 8].

Multiple mechanisms are involved in the biogenesis of EVs: exosomes originate as intraluminal vesicles (ILVs) via inward budding of the limiting membrane of maturing endosomes, giving rise to multivesicular endosomes (MVEs) [5]. MVEs are prone to fuse with lysosomes for degradation of their contents, however, they can also dock and fuse with the plasma membrane to release ILVs into the extracellular space [5]. One of the best-characterized mechanism of exosome biogenesis involves the recruitment of the endosomal sorting complex required for transport (ESCRT) machinery to ubiquitinated proteins in the early endosome. There are four ESCRT complexes (ESCRT-0, −I, −II, and -III), which associate with ESCRT-associated accessory proteins, such as the ATPase VPS4, its cofactor VTA-1, TSG101, and Alix. ESCRT-0 complex recognizes and sequesters ubiquitinated proteins on the outside of the endosomal membrane. ESCRT-I and –II complexes are responsible for starting and driving intraluminal membrane budding. ESCRT-III complex performs vesicle scission to form MVEs [9]. Trajkovic et al. have also described an ESCRT-independent exosome biogenesis pathway, which is mediated by the sphingolipid ceramide [10]. Ceramide is produced from the hydrolysis of sphingomyelin by neutral sphingomyelinase 2 (nSMase2) [10]. The cone-shaped structure of ceramide stimulates the negative membrane curvature to facilitate the membrane invagination of ILVs [10]. The authors reported that nSMase2 is needed for the release of proteolipid protein (PLP) from Oli-neu cells [10]. In addition, the ceramide-mediated exosome biogenesis pathway appears to be important for microRNA (miRNA) export via exosomes [11]. Tetraspanin CD63 has also been shown to be involved in the sorting of melanocyte protein PMEL into exosomes in an ESCRT-independent mechanism [12]. Some of the key regulators of MVE docking and fusion with the plasma membrane include several Rab family members (Rab11, Rab35, Rab27) as well as synaptotagmin-7 [13–17]. It was previously reported that cortactin and Rab27a coordinate to stabilize branched actin networks to allow MVE docking near the plasma membrane and exosome secretion at invadopodia [18].

MVs are formed by the outward budding, fission of the plasma membrane, and release into the extracellular space [5, 7]. The biogenesis of MVs is distinct from that of MVEs-derived exosomes [5, 7]. During MVs generation, there are molecular rearrangements at the sites of MVs budding resulting in an alteration of the lipid and protein compositions of the plasma membrane [5, 7]. One of the mechanisms of the MVs formation involves phospholipid reorganization by aminophospholipid translocases (floppases and flippases) [5, 7]. The translocation of phosphatidylserine (PS) from the inner leaflet to the outer leaflet by floppase induces the budding and release of MVs [5, 7]. Another contributor to MVs budding is the small GTPase protein, ADP-ribosylation factor 6 (ARF6). ARF6 stimulates phospholipase D (PLD), which subsequently leads to the association of extracellular signal-regulated kinase (ERK) with the plasma membrane [19]. ERK is responsible for the phosphorylation of myosin light-chain kinase (MLCK) [19]. Activated MLCK promotes the phosphorylation and the activation of the myosin light chain. The end result of this signaling cascade is the contraction of actomyosin at the “necks” of MVs, which facilitates MVs release [19]. Another mechanism of MVs formation is mediated by Arrestin 1 domain-containing protein 1 (ARRDC1). Nabhan et al. reported that ESCRT-I subunit TSG101 is recruited to the plasma membrane through its interaction with ARRDC1, which is dependent on a conserved PSAP motif in ARRDC1 [6]. The budding of ARRDC1-mediated microvesicles (ARMMs) needs both TSG101 and the ESCRT-associated ATPase VSP4 [6]. The association of ARRDC1 with ubiquitin ligase WWP2 subsequently leads to the ubiquitination of ARRDC1 and drives the budding of ARMMs [6]. One characteristic of ARMMs is that they differ from other MVs in their size. ARMMs are ~ 100 nm in diameter, which is similar to the size of exosomes [6]. Wang et al. recently reported that a functional NOTCH2 receptor is released via ARMMs. Once the NOTCH2-containing ARMMs are transferred to recipient cells, the expression of NOTCH2 target genes (HES1 and HES5) was induced [20].

It is now clear that EVs serve as vehicles for bidirectional communication between cells. The receptors and ligands found on the outside of EVs provide a vectorial cargo transfer to cells expressing the cognate ligand/receptors, conferring specificity to this interaction [8, 12]. There are multiple processes by which EVs and their cargoes can be transferred to recipient cells. EVs may anchor at the plasma membrane of a target cell [21, 22]. Bound EVs may fuse directly with the plasma membrane of the recipient cell [21, 22]. Additionally, bound EVs can be taken up by phagocytosis, macropinocytosis, lipid raft-mediated endocytosis, clathrin-mediated endocytosis, or caveolin-mediated endocytosis [21, 22]. When endocytosed, EVs can be targeted to lysosomes for degradation [21, 22]. An alternative fate is that EVs could fuse with the delimiting membrane of an endocytic compartment, which subsequently allows for the release of EV content into the cytosol of the recipient cells [21, 22]. EVs carry bioactive molecular cargoes, including various proteins, lipids and nucleic acids (DNA, mRNA fragments, miRNA, small nucleolar RNA, Y RNA, mitochondrial RNA, and other non-coding RNAs) that can affect the functions and phenotypes of recipient cells by altering gene expression via *de-novo* translation and/or post-translational modifications of target mRNAs [5, 8] or by activating various signaling pathways [8, 22].

Given the lack of standardized nomenclature and isolation protocols for extracellular vesicles, we will commonly refer to exosomes, microvesicles, oncosomes, or microparticles as extracellular vesicles.

## Extracellular vesicles as modulators of the tumor microenvironment

**Fig. 1:**
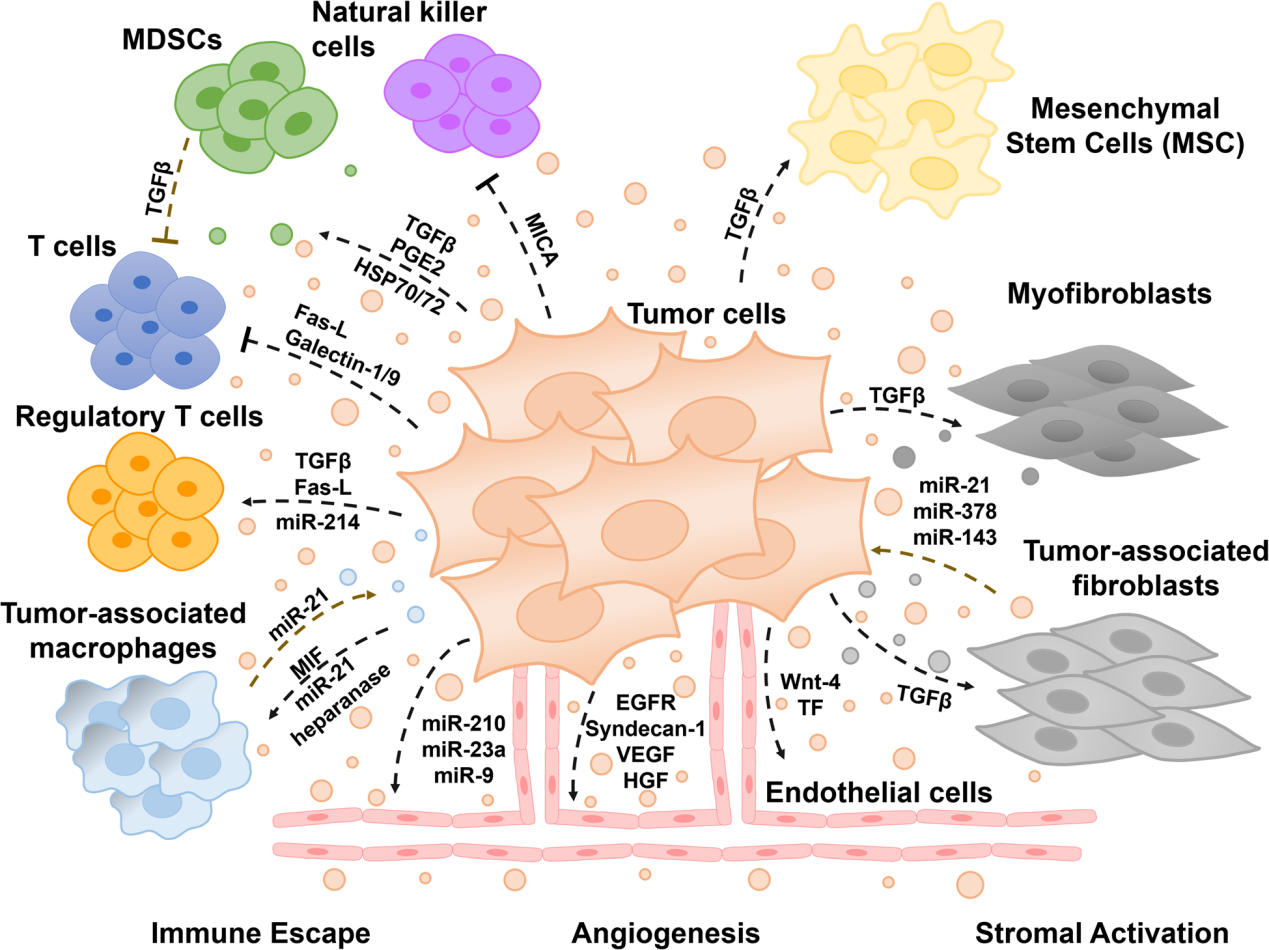
Role of the extracellular vesicles-mediated intercommunication in tumor development and progression. Tumor and stromal cells release extracellular vesicles as a mean of communication contributing to the complexity and heterogeneity of the tumor microenvironment. Extracellular vesicles-mediated transport of bioactive materials can induce a tumor microenvironment favorable for tumor growth and resistance to anti-cancer drugs

A critical biological feature that contributes significantly to cancer progression, invasion and metastasis is the ‘tumor microenvironment’ [23]*.* The tumor microenvironment (TME) is an interactive cellular environment surrounding the tumor whose main function is to establish cellular communication pathways supporting tumorigenesis [24]. The cellular component of the TME mainly comprises immune and inflammatory cells, stromal fibroblasts, and endothelial cells forming the blood vessels that secrete a series of extracellular/angiogenesis signaling molecules, which in turn lead to a functional modulation of TME [23]*.* The TME then converts into a pathological entity that continually evolves to aid cancer progression and invasion [24]*.* The extracellular vesicles (EVs) secreted by tumors, commonly known as tumor-derived EVs, have been well documented to modulate the tumor microenvironment **(**Fig. 1) [25]*.* EVs are highly specialized entities of communication carrying several surface markers and signaling molecules, oncogenic proteins and nucleic acids that can be transferred horizontally to the stromal target cells and condition the tumor microenvironment for an improved tumor growth, invasion, and metastasis [26–28]. The role of EVs in cancer progression and metastasis is described in detail below.

### Extracellular vesicles and stromal activation

Stromal cells, together with extracellular matrix components are critical components of the tumor microenvironment, playing crucial roles in tumor initiation, progression, and metastasis [29]. One of the main stromal changes within the TME is the appearance of cancer-associated fibroblasts (CAFs) [29]. CAFs constitute a major portion of the reactive tumor stroma and play a crucial role in tumor progression. Tumor-derived EVs are essential mediators of the intercommunication between tumor and stromal cells, contributing to stromal support of tumor growth. Tumor-associated EVs have been reported to play a significant role in the differentiation of fibroblasts into CAFs, inducing a tumor-promoting stroma [30]*.* In addition to fibroblasts activation, tumor-derived EVs can also induce the differentiation of mesenchymal stem cells, and other bone marrow-derived cells to become tumor-supportive cells by delivering growth factors, such as transforming growth factor-beta (TGF-β) and various miRNAs [1, 31]. For instance, breast cancer and glioma cells are capable of conferring cancer transformed characteristics to normal fibroblasts and epithelial cells through the transfer of cancer cell-derived EVs carrying the cross-linking enzyme tissue transglutaminase (tTG)-crosslinked fibronectin [32]. More recently, it was reported that ovarian cancer cells secrete EVs capable of modulating fibroblasts behavior towards a CAF-like state. The secretome of the CAFs is, in turn, able to promote the proliferation, motility, and invasion of the tumor and endothelial cells [33]. Furthermore, in a prostate cancer cell model, the release of TGF-β1-associated EVs triggers fibroblast differentiation into a myofibroblast phenotype supporting angiogenesis in vitro and accelerating tumor growth in vivo [34]. Likewise, EVs derived from osteosarcoma cells carry a high level of surface-associated TGF-β1, which induces mesenchymal stem cells to secrete interleukin-6 and is associated with increased metastatic dissemination [35]. Breast cancer cells-derived EVs have also been reported to promote the acquisition of myofibroblast-like features in mesenchymal stem cells derived from adipose tissue [36]. Moreover, colorectal cancer-derived EVs were able to induce a tumor-like behavior in mesenchymal stromal cells, suggesting that the inflammatory microenvironment initiated by cancer cells-derived EVs promotes tumor growth and invasiveness [37]. Another mechanism described in tumor-stromal remodeling via EVs is the transfer of non-coding oncogenic miRNAs. Indeed, transfer of the pro-metastatic miRNA, miR-9, in breast cancer-derived EVs enhanced the switch of human breast fibroblasts to CAFs, resulting in enhanced cell motility [38]. Consequently, CAF-derived EVs may, in turn, support tumor growth, survival, invasion, and metastasis. For instance, CAF-derived EVs have been reported to increase the expression of Snail in recipient pancreatic ductal adenocarcinoma cells and thus to promote proliferation and drug resistance [39]. It has also been shown that fibroblasts secrete EVs that promote breast cancer cells protrusive activity, motility, and metastasis by activating autocrine Wnt-planar cell polarity (PCP) signaling [40]. Moreover, breast cancer cells exposed to CAF-derived EVs carrying miRs − 21, −378e, and − 143 display significantly increased stemness and EMT phenotypes [41].

### Extracellular vesicles and angiogenesis

Angiogenesis is the process of new blood vessels formation from pre-existing vessels occurring in physiological conditions such as growth or in response to tissue injury. In healthy tissues, angiogenesis is tightly regulated by a precise balance between stimulatory and inhibitory angiogenic signals controlling the proliferation and migration of endothelial cells. An imbalance in this regulatory network may cause several diseases, such as cancer. In the past decade, EVs secreted by different cells within the tumor microenvironment have been shown to be important mediators of pathological angiogenesis through the release of angiogenic factors that can be transferred to endothelial cells, thus leading to the generation of a pro-angiogenic niche that supports tumor growth [42]. Indeed, EVs produced by human lung or colorectal cancer cells transfer oncogenic EGFR to cultured endothelial cells, in which they elicit EGFR-dependent responses, including activation of MAPK and AKT pathways, as well as an autocrine production and signaling of VEGF [43]. It was also reported that upregulation of heparanase in myeloma and breast cancer cells is associated with increased release of Syndecan-1, Vascular Endothelial Growth Factor (VEGF), and Hepatocyte Growth Factor (HGF) in EVs, leading to increased endothelial invasion through the ECM [44]. Furthermore, hypoxic glioblastoma cells have been shown to induce the secretion of high amounts of tissue factor (TF)-associated EVs that trigger a paracrine activation of endothelial cells through a PAR-2-mediated heparin-binding EGF signaling [45]. Interestingly, a study conducted by Kucharzewska and colleagues has shown that endothelial cells were programmed by glioblastoma cell-derived hypoxic EVs to secrete several potent growth factors and cytokines and to stimulate pericyte PI3K/AKT signaling activation and migration. Using an in vivo glioblastoma mouse xenograft model, the authors found that hypoxic EVs significantly enhanced tumor vascularization, pericyte vessel coverage, and glioblastoma cell proliferation [46]. Also, hypoxic colorectal cancer cells have been shown to secrete Wnt4-enriched EVs that promote beta-catenin (β-catenin) nuclear translocation and proliferation of endothelial cells [47]. Tumor EVs have also been reported to modulate angiogenesis in tumors via the release of non-coding RNAs. For example, miR-9 contained in EVs promotes tumor angiogenesis and endothelial cells migration through the reduction of suppressor of cytokine signaling 5 (SOCS5) levels and the activation of the JAK/STAT pathway [48], while EVs carrying miR-23a are capable of inducing angiogenesis in different angiogenic model systems by targeting SIRT1 in recipient endothelial cells [49]. Likewise, neutral sphingomyelinase 2 (nSMase2) (an enzyme that generates ceramide) promotes angiogenesis in endothelial cells through the transfer of pro-angiogenic EVs enriched for miR-210 [50].

### Extracellular vesicles and immune escape

The tumor microenvironment is infiltrated by a variety of immune cells, such as lymphocytes (T cells, B cells, natural killer cells, and T regulatory cells), dendritic cells, monocytes, macrophages, myeloid-derived suppressor cells (MDSC), and granulocytes (neutrophils, basophils, eosinophils, and mast cells). The main role of these cells is to assure immune surveillance. However, tumor cells have been recognized to be capable of modulating signaling pathways within these immune cells and converting them into an immunosuppressive entity, thus leading to enhanced cancer cell survival and proliferation [51]. Despite the fact that EVs contain tumor antigens capable of priming an anti-tumor immune response, accumulating evidence demonstrates that tumor cells utilize EVs in order to suppress the anti-tumor response through the secretion of bioactive immunosuppressive molecules. Actually, EVs have been shown to be critical mediators of the immune-cancer cell communication. One example of how tumor-derived EVs aid in evading immune surveillance is by inducing apoptosis in immune cells. Indeed, several tumor-derived EVs have been shown to be enriched for Fas ligand (Fas-L) which induces cell apoptosis when binding to its receptor. Wieckowski et al. described that Fas-L-positive tumor-derived EVs induce immune suppression by promoting the expansion of T regulatory cells and the apoptosis of anti-tumor CD8(+) effector T cells, thus contributing to immune escape [52]. Similarly, this immune suppression mechanism through the release of Fas-L-containing EVs capable of inducing T-cell apoptosis was also reported in several cancer models, such as head and neck squamous cell carcinoma, melanoma, prostate, and colorectal cancer [53–56]. The presence of other mediators of T- cell apoptosis in tumor-derived EVs has been reported for galectin-1 and -9, both causing T-cell apoptosis and immune suppression [57, 58]. Similarly, EVs released from mesothelioma, acute myeloid leukemia, or colorectal cancer have been shown to contain the transforming growth factor (TGF-β) on their surface and to deliver it to T-cells, inhibiting their proliferation in response to interleukin-2 and changing their phenotype to regulatory T cells [59–61]. Moreover, tumor-released EVs have been shown to impair monocyte differentiation into dendritic cells and to promote the generation of a TGF-β secreting myeloid immunosuppressive cell subset (MDSC), which inhibit T lymphocyte proliferation [62]. The enrichment of prostaglandin E2 (PGE2) and TGF-β in tumor-derived EVs induces the accumulation of MDSCs with immune suppressive properties [63]. Similarly, it has been shown that tumor-derived EV-associated Hsp72 or Hsp70 mediate the suppressive activity of the MDSCs via STAT3 activation [64, 65]. The presence of HSP72 and HSP105 in EVs has also been reported in melanoma, lung, and breast cancer cell lines, as well as in the serum of breast cancer patients. These EVs have been shown to activate dendritic cells and induce secretion of interleukin-6, which promotes tumor invasion by increasing MMP-9 metalloproteinase expression [66]. Tumor cells can also release EVs containing MHC class 1 related chain ligand A (MICA) that is capable of binding to the NK cells receptor, NKG2D, leading to its downregulation and resulting in a marked reduction in NK cytotoxicity independent of NKG2D ligand expression by the target cell [67]. Tumor-released miRNAs have also been involved in immune suppression. For instance, miR-214 secreted into EVs from Lewis Lung carcinoma cells was sufficiently delivered into recipient T cells and in vivo studies indicated that miR-214 mediates regulatory T cell expansion resulting in enhanced immune suppression and tumor growth in mice [68].

### Extracellular vesicles and metastasis

Metastasis is a multistep process leading to the dissemination of primary tumor cells to distant organs. Tumor-derived EVs have almost been involved in all steps of tumor invasion and metastasis [15, 69–71]. Studies have reported that tumor-associated EVs play a significant role in invasion and metastasis through invadopodia formation [18, 72]. Invadopodia are dynamic actin-rich membrane protrusions that tumor cells produce to degrade and invade through the extracellular matrix [72]. It was recently proposed that invadopodia are docking sites for EVs facilitating the degradation of the extracellular matrix through a localized secretion of metalloproteinase MT-1-MMP, thus promoting cell invasion [15, 73]. Similarly, the migration of tumor cells through tissues and chemotactic gradients is also initiated by the formation and release of fibronectin-bound EVs at the leading edge of migrating cells. These fibronectin-bound EVs are proposed to promote adhesion assembly and stabilization allowing a directional and persistent tumor cell migration [74, 75]. Tumor-derived EVs are also known to influence the integrity of vascular barriers, which is frequently associated with metastatic dissemination. Proteomics analysis of tumor-associated EVs has shown that EVs release a number of proteins such as SERPINA1, SERPINF2, and MMP9, the up-regulation of which play a significant role in ECM remodeling, vascular leakiness, and invasiveness [76]. Likewise, melanoma-derived EVs have been shown to induce pulmonary vascular leakiness [77], while EVs produced by glioblastoma cells containing high levels of VEGF-A induce endothelial cell permeability and angiogenesis in vitro [78]. In addition, EVs derived from lung cancer or breast cancer cells were reported to carry miR-23a and miR-105 respectively, which both target tight junction protein ZO-1, thereby increasing vascular permeability and cancer transendothelial migration [79, 80]. An interesting feature of tumor-derived EVs is their ability to establish a pre-metastatic niche, a phenomenon where the primary tumor can promote its own metastasis by recruiting stromal cells to distant organs or by modulating gene expression of distant cells in order to establish a growth supportive environment. EVs derived from colorectal cancer cells enriched for miR-21 can be specifically targeted to liver tissue and induce liver macrophage polarization towards an interleukin-6 (IL-6)-secreting pro-inflammatory phenotype, therefore promoting an inflammatory pre-metastatic niche supportive of liver metastasis [81]. Moreover, melanoma EVs were shown to home to lymph nodes and consequently enhance the migration of melanoma cells to sentinel lymph nodes. In addition, melanoma EVs were able to upregulate the expression of genes within the distal lymph node microenvironment related to tumor cell recruitment to sentinel nodes, extracellular matrix modifiers promoting trapping of melanoma cells, and vascular growth factors promoting melanoma growth, creating a pre-metastatic niche supportive of metastasis [82]. Melanoma-derived EVs were further reported to educate bone marrow-derived cells towards a pro-vasculogenic and pro-metastatic phenotype through the receptor tyrosine kinase MET [77]. More recently, the uptake of pancreatic ductal adenocarcinoma-derived EVs by Kupffer cells (liver macrophages) was reported to cause TGF-β secretion and upregulation of fibronectin production by hepatic stellate cells, leading to an enhanced recruitment of bone marrow-derived macrophages through macrophage migration inhibitory factor (MIF), whose association with EVs correlated with liver metastasis occurrence and disease progression [83]. Interestingly, using different tumor models, Hoshino and colleagues reported that the metastatic organotropism and establishment of a pre-metastatic niche is mediated by EVs via the secretion of different sets of integrins (for e.g. integrin- α6β4, α6β1, or αvβ5) that favor the preferential fusion of tumor cells with resident cells at their predicted destination. The authors showed that tumor-derived EVs taken-up by organ-specific cells prepared the pre-metastatic niche and that distinct integrin patterns predicted the organotropism of tumor cells, integrins α6β4, and α6β1 being associated with lung metastasis, while integrin αvβ5 was found to be associated with liver metastasis [84]. Reprogrammed glucose metabolism is a hallmark of cancer cells. Remarkably, cancer cells are also proficient in reprograming the glucose metabolism of stromal cells through the release of EVs carrying high levels of the miR-122 that target the glycolytic enzyme pyruvate kinase. This mechanism is proposed to facilitate metastasis by increasing nutrient availability in the pre-metastatic niche [70].

## Extracellular vesicles as modulators of anti-cancer drug resistance

**Fig. 2:**
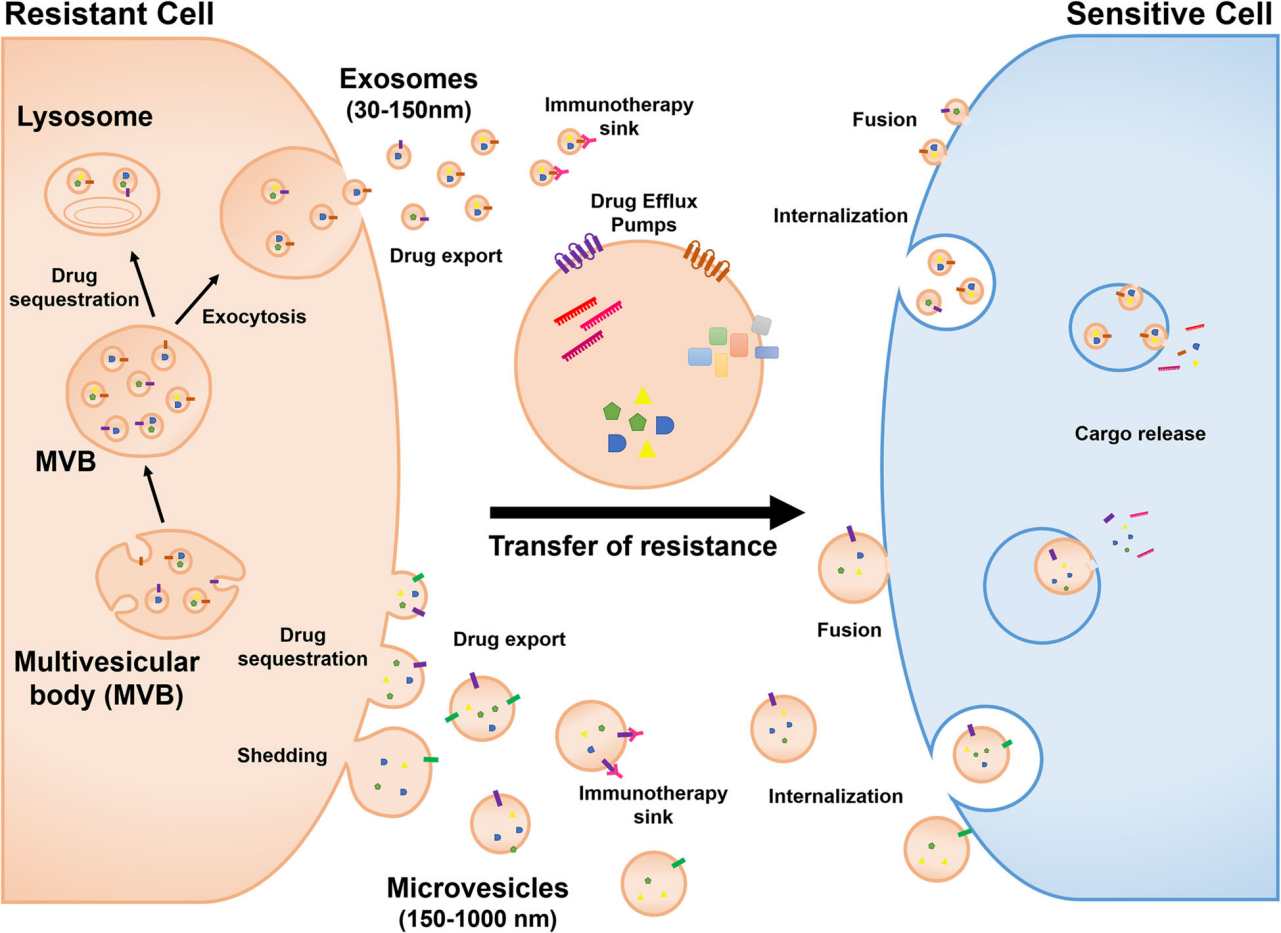
Mechanisms of extracellular vesicles-mediated transfer of anti-cancer drug resistance. Extracellular vesicles can mediate drug resistance by directly exporting or sequestering cytotoxic drugs reducing their effective concentration at target sites. Extracellular vesicles can also compete with bona fide target cells for the binding of immunotherapeutic agents targeting cellular antigens. Extracellular vesicles also mediate transfer of drug resistance to drug-sensitive cancer cells through the horizontal transfer of specific bioactive cargoes including drug efflux pumps, prosurvival factors, inhibitors of apoptosis, and non-coding RNAs

Drug resistance poses a serious challenge for the treatment of cancer and occurs when cancer cells become tolerant to anti-cancer drugs. Although many types of cancers are initially susceptible to anti-cancer drugs, tumor cells can develop resistance over time through different mechanisms that impair drug efficacy. The most common mechanisms of drug resistance include genetic or epigenetic upregulation of prosurvival signaling and inhibition of apoptotic pathways, drug inactivation or alteration of drug target molecules, overexpression of multidrug resistance proteins (MDR) and increased transport of efflux pumps, or drug export. Recently, the emergence of EVs as novel drug resistance modulators has added to the complexity of resistance mechanisms. EVs mediate intercellular communication by transferring proteins and nucleic acids to remote target cells. The development of drug resistance via EVs is articulated around mechanisms involving such cargo. EVs can mediate drug resistance by directly exporting or sequestering cytotoxic drugs, reducing their effective concentration at target sites. Recent evidence has shown that EVs play an important role not only in mediating drug resistance, but also in conferring resistance to drug-sensitive cancer cells. Indeed, EVs are capable of horizontal transfer of specific bioactive cargoes that may alter cell cycle control and apoptotic programs in recipient cells **(**Fig. 2). EVs can also mediate intercommunication between cancer cells and stromal cells within the tumor microenvironment, leading to the acquisition of drug resistance and tumor progression. Mastering our understanding of these resistance mechanisms will help in improving cancer treatments and subsequently patients’ outcome. Detailed mechanisms by which resistance may occur are outlined in this section.

### Extracellular vesicles as a sink for immunotherapies

Cancer cells use extracellular vesicles to compromise targeted therapies. EVs carry on their surface, a plethora of cellular antigens displayed in an orientation identical to that found on the surface of cells from which they emanate. The presence, on EVs surface, of cellular antigens targeted by immunotherapy acts as a sink for monoclonal antibodies-based drugs, thereby diminishing their bioavailability to their intended target. In the case of B-cell lymphoma, the presence of CD20 on the surface of EVs protects targeted lymphoma cells from rituximab (an anti-CD20 monoclonal antibody) attack [85]. In vitro as well as in vivo studies in breast cancer point to the role of HER2-positive extracellular vesicles in modulating resistance to anti-HER2 monoclonal antibody Trastuzumab. Extracellular vesicles secreted either by HER2-positive tumor cells in vitro or found in the serum of breast cancer patients bind to Trastuzumab, and inhibit its activity in vitro [86]. More recently, EVs have been involved in another type of drug resistance mediated by cellular receptor expression. The immunotherapy breakthrough crowned by the 2018 Medicine Nobel prize consists in the use of inhibitors of immune-checkpoints to unleash the power of an immune system otherwise tamed by immune checkpoint ligand expressed on the surface of tumor cells. The disruption of the interaction of the checkpoint ligand (e.g. PD-L1) with the inhibitory checkpoint receptor (PD-1) on T cells, restores T cell function and anti-tumor immunity. However, not all patients respond to such immune checkpoint inhibitor therapy, and the presence of checkpoint ligand (PD-L1) on EVs early after therapy, classifies patients as responders or resistant to anti-PD-1 therapy in melanoma [87]. By capturing the immunotherapeutic antibody on their surface, EVs drive this antibody away from the tumor, leaving it free to engage PD-1 on oncoming tumor-specific T cells. The same mechanism has been described in the case of glioblastoma in vitro, in which tumor-derived EVs were shown to express PD-L1 and inhibit T cell proliferation as well as antigen-specific T cell responses [88].

### Extracellular vesicles-mediated drug export and sequestration

Irrespective of the administration route of anti-cancer drugs, systemic, oral or subcutaneous, the main goal of the treatment is to target drugs to the tumor site, where cellular drug uptake or membrane permeability are determinant in the drug efficacy and treatment success. However, it is recognized that abnormal tumor architecture (e.g.: poor vascularization, abnormal extracellular matrix) complicates drug uptake and is associated with therapy failure and drug resistance [89]. Even in case of efficient uptake of drugs by targeted cells, cancer cells are known to export drugs in the extracellular space using specialized transporters of the multi-drug resistance (MDR)-ATP binding-cassette (ABC transporters) system [90]. These pumps reduce the intracellular accumulation of many anti-cancer drugs to sub-therapeutic levels, thus decreasing or abolishing drug efficacy. In addition, EVs can be utilized by cancer cells as drug vehicles to facilitate drug resistance through drug sequestration and expulsion. Shedden and colleagues were the first to report a positive correlation between the expression of genes associated with vesicle shedding and drug resistance in a large panel of different cancer cell lines [91]. Furthermore, using a breast cancer cell line, they showed by fluorescence microscopy and flow cytometry that the fluorescent chemotherapeutic agent doxorubicin was physically encapsulated into vesicles and expelled out into the extracellular media [91]. More recently, melanoma cells were shown to resist to cisplatin treatment through an extracellular acidification-mediated increase of EVs secretion and the direct export of cisplatin into these vesicles [92]. Similarly, cisplatin was found to be disposed out of resistant ovarian carcinoma cells in extracellular vesicles [93]. Interestingly, EVs from resistant cells not only contained Multidrug Resistance-associated Protein 2 (MRP-2) but also the copper-transporting P-type ATPases, ATP7A and ATP7B [93]. B-cell lymphoma cells also efficiently extruded doxorubicin and pixantrone in EVs in vitro [94]. Interestingly, ATP-transporter A3 (ABCA3) expression is crucial for EVs biogenesis and contributes to the observed drug resistance. Indeed, genetic or chemical depletion of ABCA3 enhanced intracellular retention of both drugs [94].

Cancer cells can also sequester drugs within intracellular vesicles preventing them from reaching the targeted subcellular compartment and rendering them nonfunctional. In a breast cancer cell model resistant to mitoxantrone, cells displayed increased EV-like structures at the plasma membrane containing the ATP-binding cassette ABCG2 in which mitoxantrone was significantly sequestered [95]. Subcellular drug sequestration was also shown to be mediated by ABCA3 in leukemia cells resistant to a panel of cytostatic drugs [96]. Indeed, ABCA3 localized to the limiting membranes of lysosomes and multivesicular bodies and caused cytostatic drugs to be efficiently sequestered [96].

### Extracellular vesicles-mediated transfer of drug efflux pumps

In addition to drug export or sequestration, cancer cells can transmit resistance through horizontal transfer of EVs carrying drug efflux pumps. Drug efflux transporters of the multi-drug resistance (MDR)-ATP binding-cassette (ABC transporters) system have long been acknowledged as major contributors to multidrug resistance in tumor cells [90]. EVs carrying P-glycoprotein (P-gp, MDR-1 or ABCB1), one of the most well-studied drug efflux pump, have been implicated in the transfer of multidrug resistance to sensitive cells in several human cancer models, such as prostate and ovarian cancers, acute T lymphoblastic leukemia, and osteosarcoma [97–100]. Indeed, EVs from sera of patients undergoing a course of docetaxel treatment compared to matched EVs from the same patients prior to commencing docetaxel treatment, when applied to both prostate cancer drug sensitive and resistant cells, showed a correlation between cellular response to docetaxel and patients’ response to treatment with docetaxel [97]. Similarly, extracellular vesicles-mediated intercellular transfer of functional MRP1 drug efflux transporter (ABCC1) was reported in leukemia cells [101]. Other drug efflux exporters such as ABCG2 or ABCA3 have been shown to transfer horizontally through EVs and modulate drug resistance in recipient cells [85, 102]. Although tumor cells represent an abundant source of EVs, it is important to question whether the consequences of this transfer of cargo is sustainable in vivo. The presence of selective P-gp/MDR-1 mRNA in EVs released from doxorubicin-resistant osteosarcoma cells suggests that resistant tumor cells use several means to spread drug resistance to sensitive cells, either by transferring MDR proteins directly to sensitive cells or by transferring the mRNA that encodes them, contributing to the diversity of drug resistance mechanisms [100]. On the other hand, depletion of drug efflux pumps through EVs exocytosis has been shown to increase the sensitivity of tumor cells to anti-cancer drugs in vitro. In an in vivo setting, one can legitimately wonder whether this in vitro beneficial response could not result in a net drug resistance within the tumor microenvironment. Indeed, these EVs loaded with MDR transporters could be transferred to other cells within the heterogeneous tumor itself or the stromal cells within the tumor microenvironment, therefore possibly influencing their response to treatment.

### Extracellular vesicles-mediated export of prosurvival cargo

EVs transfer of cargo can contribute to the heterogeneity of tumor response to anti-cancer drugs. This cargo includes prosurvival factors, which enhance cell viability and decrease apoptosis sensitivity, thus leading to resistance to anti-cancer drugs. For instance, components associated with the PI3K/AKT pathway, one of the major oncogenic signaling axis involved in cancer cell proliferation and survival, have been reported in EVs. In hepatocellular carcinoma (HCC) invasive cells lines, resistance to Sorafenib in vitro as well as in vivo was induced by delivery of hepatocyte growth factor (HGF) through EVs and subsequent activation of the HGF/c-MET/PI3K/AKT signaling pathway [103]. In addition, platelet-derived growth factor receptor-beta (PDGFR-β), which is enriched in EVs released by melanoma cells resistant to BRAF inhibitor, PLX4720, can be transferred to recipient melanoma cells, resulting in a dose-dependent activation of PI3K/AKT signaling and escape from BRAF inhibition [104]. More recently, triple negative breast cancer cell lines resistant to Docetaxel and Doxorubicin were shown to release EVs that induced resistance to these chemotherapy drugs in recipient non-tumorigenic breast cells [105]. Indeed, these EVs caused changes in gene expression associated with cell proliferation and apoptosis including the PI3K/AKT pathway, suggesting that they may contain ligands or receptors connected to the PI3K signaling axis [105]. Likewise, EVs can also carry prosurvival molecules that modulate the immune system functions likely inducing immune tolerance and escape. Transforming growth factor-β (TGF-β) cytokines have been shown to play a critical role in establishing immunological suppression [106]. Indeed, TGF-β1 was found in tumor-derived EVs and reported to inhibit the proliferation of healthy donor peripheral blood lymphocytes in response to IL-2 and to induce regulatory T cells [59]. Additionally, in vivo and in vitro studies on HER2-overexpressing breast cancer have reported the presence of increased amounts of the immunosuppressive cytokine TGF-β1 in EVs released from cells resistant to HER2-targeting drugs [107]. Although the patients’ cohort was too small to be conclusive, these findings suggest that the level of EVs-associated TGF-β1 in the plasma of the patients correlates with resistance to Lapatinib and Trastuzumab [107]. Resistance to apoptosis is a vital escape mechanism by which tumor cells acquire drug resistance and thus contribute to cancer progression. EVs-mediated delivery of prosurvival factors is proposed to provide tumor cells with an additional mechanism to suppress cell death induced by anti-cancer drugs. Survivin is a prosurvival protein member of the inhibitors of apoptosis (IAP) family shown to be present in EVs derived from different tumor types [108–110]. Survivin has been implicated in the suppression of cell death and the regulation of mitosis, and therapeutic strategies targeting survivin in cancer are intensively investigated [111]. Indeed, Khan and colleagues identified EVs as mediators of stress-induced survivin secretion from HeLa cells treated with a sublethal dose of proton irradiation [109]. More recently, Kreger and colleagues have reported that treating highly aggressive MDA-MB-231 breast cancer cells with Paclitaxel (PTX) induces the secretion of EVs enriched with survivin that significantly promote the survival of serum-starved and PTX-treated fibroblasts and SKBR3 breast cancer cells [112].

Moreover, the enrichment of microRNAs (miR) in EVs have been shown to promote anti-cancer drugs resistance in different cancers **(**Table 1**)**. For example, the investigation of drug resistance in breast cancer cells or pancreatic ductal adenocarcinoma cells revealed that EVs-mediated transfer of miR-155 to sensitive cells resulted in chemoresistance spreading. Interestingly, increased accumulation of miRNA in EVs exposed to chemotherapeutic agents can also serve as a disposal mechanism aimed at decreasing the intracellular levels of miRNA with drug sensitivity promoting roles [113, 114].

**Table 1:** Extracellular vesicles miRNA cargo and chemoresistance in different cancers

Cancer	Anti-cancer drugs	Cell lines	miRNA cargo	Mechanism	Reference
Lung	Cisplatin	A549/A549-DDP	↓ miR-100-5p	horizontal transfer	[133]
Lung	Gemcitabine	A549/A549-GR	↑ miR-222-3p	horizontal transfer	[134]
Lung	Cisplatin	A549/H1299	↑ miR-96	horizontal transfer	[135]
Lung	Cisplatin	A549/A549-DDP	↓ miR-146a-5p	horizontal transfer	[136]
Breast	Docetaxel	MCF-7	↑ miR-100, miR-222, miR-30a, miR-17	horizontal transfer	[137]
Breast	Tamoxifen	MCF-7	↑ miR-221/222	horizontal transfer	[138]
Breast	Cisplatin 17-AAG PU-H71	Hs578Ts	↓ miR-134	horizontal transfer	[139]
Breast	Doxorubicin Paclitaxel	MCF-7/MDA-MB-231	↑ miR-155	horizontal transfer	[140]
Breast	Docetaxel Epirubicin Gemcitabine	MDA-MB-231/HMLE	↑ miR-1246	horizontal transfer	[141]
Breast	Adriamycin	MCF-7/MCF-7-Adr	↑ miR-222	horizontal transfer	[142]
Oral cavity	Cisplatin	HSC-3/HSC-3R SCC-9/SCC-9R	↑ miR-21	horizontal transfer	[143]
Melanoma	Vemurafenib (PLX4032)	MML-1/MML-1R A375 PDX	↑ miR-211–5p	Autocrine	[144]
Glioblastoma	Temozolomide	SHG-44/U87MG	↑ miR-221	horizontal transfer	[145]
Prostate	Docetaxel	22Rv1/22Rv1RD DU145/DU145RD PC3/PC3RD	↓ miR-34a	horizontal transfer	[146]
Colon	Fluorouracil (5-FU)	DLD-1/DLD-1-5-FU	↑ miR-145, miR-34a	expulsion	[113]
Pancreas	Gemcitabine	Panc1/Panc1-GR	↑ miR-155	horizontal transfer	[147]
Leukemia	Imatinib	K562/K562-G01	↑ miR-365	horizontal transfer	[148]
Leukemia	Daunorubicin	HL60/HL60AR	↑ miR-196, miR20a	expulsion	[114]

### Tumor microenvironment-mediated intercellular communication and drug resistance

Tumor growth and drug resistance are not only determined by cancer cells but are also supported by non-tumor cells within the tumor microenvironment. The importance of the role of EVs in the intercellular communication within the tumor microenvironment is increasingly acknowledged. The bidirectional EV-mediated transfer of cargo to and from non-tumor cells effectively influences recipient cell’s phenotype as well as their response to anti-tumor treatments, thus promoting the development of an environment hospitable towards cancer growth, invasion, and metastasis. For instance, by secreting chemoresistance-inducing EVs containing Snail and miR-146, pancreatic cancer-associated fibroblasts (CAFs), that are intrinsically resistant to the chemotherapeutic agent gemcitabine, have been shown to mediate the transfer of resistance to pancreatic cancer epithelial cells when exposed to this drug, thereby increasing their proliferation and survival [39]. Similarly, Binenbaum and colleagues have recently reported that transfer of miR-365 in macrophage-derived EVs induces resistance of pancreatic adenocarcinoma cells to gemcitabine in vitro and in vivo [115]. Moreover, CAF-derived EVs further promoted tumor growth of colorectal cancer stem cells (CSCs) upon treatment with 5-fluorouracil or oxaliplatin, even though these cells were intrinsically chemoresistant. Interestingly, the authors have also shown that inhibition of EVs secretion by CAF increased chemosensitivity of colorectal CSCs [116]. Likewise, the vesicular transfer of miR-21 from cancer-associated adipocytes and fibroblasts to ovarian cancer cells has been reported to decrease apoptosis and promote chemoresistance to paclitaxel by downregulating the expression of apoptotic peptidase activating factor (APAF1) mRNA [117]. Similarly, tumor-associated macrophages (M2 polarized macrophages)-derived secretion of miR-21 has been shown to confer cisplatin resistance in gastric cancer cells. Functional studies revealed that vesicular miR-21 can be directly transferred from macrophages to gastric cancer cells, where it suppresses cell apoptosis and enhances activation of PI3K/AKT signaling pathway through down-regulation of PTEN [118]. Furthermore, Boelens and colleagues have previously reported that vesicular RNA from stromal cells, which are largely noncoding transcripts and transposable elements, can be transferred to breast cancer cells, leading to the expansion of therapy and radiation resistant breast cancer cells through a mechanism involving NOTCH3 induction [119]. Accumulating pieces of evidence show that mesenchymal stem cells (MSCs) are chemo-attracted by tumors where their plastic properties are reported to support tumor growth. Indeed, human MSC-derived EVs were found to induce resistance of gastric cancer cells to 5-Fluorouracil both in vivo and ex vivo through the inhibition of 5-fluorouracil-induced apoptosis and enhanced expression of multidrug resistance-associated proteins. The authors have reported that mesenchymal stem cells-EVs could induce drug resistance in gastric cancer cells by activating CaM-Ks/Raf/MEK/ERK signaling pathway [120].

Cancer and stromal cells within the tumor microenvironment have often restricted access to nutrients and oxygen and thus are subjected to hypoxia [121]. In this setting, hypoxia-induced EVs have been shown to contribute to the chemoresistance of ovarian cancer cells in a mechanism involving STAT3. Indeed, hypoxia-induced EVs are capable of increasing the survival of tumor cells in response to cisplatin treatment in vitro. In addition, cisplatin efflux through EVs was shown to be significantly augmented in ovarian cancer cell lines cultured under hypoxic conditions [122].

The crosstalk between tumor cells and stromal cells is bidirectional as cancer cells can also influence the behavior of stromal cells through EVs secretion. For instance, Bandari and colleagues found that anti-myeloma chemotherapy (Bortezomib, Carfilzomib, or Melphalan) dramatically stimulates surface heparanase-rich EVs secretion capable of degrading the ECM and that exposure of these EVs to macrophages enhanced the secretion of TNF-α (an important myeloma growth factor) and stimulated their migration [123]. On the other hand, anti-cancer drugs (Paclitaxel, etoposide, irinotecan hydrochloride, or carboplatin) have been reported to cause chemoresistant hepatocellular carcinoma cells to release EVs that elicit superior anti-tumor NK cell responses compared to chemosensitive cells, in a mechanism mediated by EV secretion of heat shock proteins. Interestingly, this study provides a clue for finding an effective vaccine for hepatocellular carcinoma immunotherapy [124].

### Strategies to mitigate EVs-mediated drug resistance

When considering strategies to mitigate the role of EVs in transferring drug resistance, two major avenues come to mind. The first one is to modulate the production of EVs, by blocking their secretion. Because of the universality of EVs secretion and of the lack of drugs that can specifically target secretion of EVs by cancer cells, this strategy is likely to interfere with unwanted EVs secretion, including the secretion of EVs involved with the acquisition and transfer of resistance to anti-cancer drugs, as well as with the secretion EVs involved in normal physiological processes. The second possibility for mitigating drug resistance mediated by tumor-derived EVs is to specifically remove these EVs once they have been produced, without interfering with EVs secretion. This approach has the advantage of maintaining the secretion of “beneficial” EVs, affecting only those EVs secreted by cancer cells. This second approach relies on the availability of markers specific for tumor-derived EVs. Such markers are available for certain cancers. Both strategies have been pursued in vitro and in vivo.

Federici et al. described the effect of proton pump inhibitor on both cisplatin uptake and EVs release in vitro an in vivo in a mouse xenograft model of melanoma, in which they show that treatment with a proton pump inhibitor decreases the overall EVs release and increases tumor cell sensitivity to cisplatin [92]. Roseblade et al. have evaluated the efficacy of several inhibitors of EVs release in response to calcium mobilization, including the use of a calpain inhibitor [125], which was also shown to block EVs release by prostate cancer cell lines in vitro and increased their sensitivity to chemotherapy in vivo [126]. Similarly, Muralidharan-Chari et al. showed that the inhibition of EVs release by preventing the activation of the extracellular signal-regulated kinase (ERK) using a MEK inhibitor, resulted in an increased sensitivity of pancreatic cancer cell lines to gemcitabine in vitro and in a tumor graft model in vivo [127]. While the selectivity of agents specifically blocking EVs release in cancer maybe lacking for most, some inhibitors target isoforms of enzymes preferentially associated with cancer cells. This is the case for inhibitors of peptidylarginine deiminase PAD2 and PAD4 which are overexpressed in prostate, ovarian and other types of cancer cells, and whose inhibition by chloramidine reduces the release of EVs and increases cancer cell sensitivity to drugs [128]. In a more systematic in vitro approach, interference with different steps of EVs biogenesis in prostate and breast cancer cell lines, Kosgodage et al. confirmed that among a series of 11 inhibitors targeting various steps of EVs biogenesis, PAD inhibitors, as well as inhibitors of PKC (Bisindolylmaleimide-I), were the most powerful inhibitors in prostate and breast cancer cell lines [129]. Recently, the same group demonstrated the powerful role of cannabinol (CBD) as an inhibitor of EVs release by prostate, hepatocellular carcinoma, and breast cancer cell lines, increasing cell sensitivity to anti-cancer drugs [130].

While these pharmacologic interventions have proven successful in vitro and in some cases in animal models in vivo, their lack of selectivity for cancer cells, for the most part, calls for some reservations on their systematic therapeutic use. This is not the case for the specific removal of circulating EVs from plasma. A method similar to hemodialysis, extracorporeal hemofiltration using cartridges made of hollow fibers with a size cutoff of 200 nm coupled with an affinity matrix allows the specific removal of ultrafiltrated EVs. This method, called Adaptive Dialysis-like Affinity Platform Technology (ADAPT™), has been originally developed by Aethlon Medical Inc. for removing Hepatitis C virus (HCV) particles from the blood of infected patients. The safety and efficacy of the method have been clinically validated in HCV infected end-stage renal disease patients using a lectin as an affinity matrix for the selective removal of HCV particles [131]. The extension of this method to the specific removal of EVs with a size inferior to that of the hollow fiber cutoff has been discussed by Marleau et al. [132]. In a previous section, we discussed the involvement of EVs in the escape to immunotherapies, by acting as a sink for immuno-targeting drugs specific for cancer-associated antigens such as CD20 in the case of B cell lymphoma [85], HER2 in the case of breast cancer [86] and more generally, PD-L1 [87, 88]. The specific removal of EVs expressing these antigens by an appropriate affinity hemofiltration device, such as those described in the ADAPT™ method will likely mitigate the immunotherapy sink effect mediated by EVs bearing the targeted antigens.

## Conclusions and future perspectives

Drug resistance is a huge hurdle in the treatment of cancer. Among the mechanisms governing the establishment of resistance to anti-cancer therapies, EVs have recently emerged as important modulators of drug resistance through a variety of mechanisms described in this review. EVs dynamically contribute, even though transiently, to the heterogeneity of the tumor through their diverse cargo content. Unraveling the precise biological composition of EVs will be critical to determining their role in cancer and will likely aid in developing therapies targeting these roles. However, the field still struggles to assess EVs heterogeneity due to the lack of standardized isolation techniques that go beyond subcellular origin, size, and floatation density. Further dissection of EVs heterogeneity will be essential to improving our understanding of the critical roles of EVs in cancer.

Exploiting EVs molecular cargo as well as the potential development of EVs as drug vehicles for effective therapeutic strategies both hold promises in cancer diagnostics and therapeutics. Omics on EVs derived from liquid biopsies (such as blood, saliva or urine) will likely aid in the early diagnosis of cancer through biomarkers discovery or in the assessment of response to therapies while avoiding invasive biopsy procedures. Related to therapeutics, EVs have been proposed as a new type of drug delivery system. Bioengineered EVs loaded with chemotherapeutic drugs or expressing ligands which target particular malignant cells have the potential for future cancer treatment. The inherent protection of the cargo and personalized cellular targeting simultaneously enhance the solubility, stability, and specificity of the therapeutic agent.

Given the prominence of EVs in almost all aspects of tumor development and progression, it seems evident to explore translational approaches that would prevent these undesirable effects. Nevertheless, EVs-mediated cell-to-cell communication is a conserved mechanism in normal cell physiology and their inhibition is likely to be toxic unless specific strategies distinguishing pathogenic EVs from beneficial ones are developed. In order to develop such strategies, it is essential to establish standardized techniques allowing consistent isolation of EVs subpopulations. This knowledge is necessary to identify cancer-derived EVs that should be targeted by any therapeutic approach. The use of EVs as cell-free therapies has also been employed in cancer vaccine and immunotherapy fields. Encouraging studies suggest the use of immune cells-derived EVs as a new potential strategy for cancer vaccine research. Only if taken together, technology and biology will pave the way for the future use of EVs in many clinical applications.

## Abbreviations

ABC: ATP binding-cassette
ADAPT™: Adaptive Dialysis-like Affinity Platform Technology
AKT: AKT serine/threonine kinase
Alix: Apoptosis-linked gene 2-interacting protein X
APAF1: Apoptotic peptidase activating factor
ARF6: ADP-ribosylation factor 6
ARMM: ARRDC1-mediated microvesicles
ARRDC1: Arrestin 1 domain-containing protein 1
ATP7A: ATPase Copper Transporting alpha
ATP7B: ATPase Copper Transporting beta
CAF: Cancer-associated fibroblast
CaM-K: Calcium/calmodulin-dependent protein kinase
CBD: Cannabinol
c-MET: MET proto-oncogene, receptor tyrosine kinase
CSF1: Colony stimulating factor 1
CXCR4: C-X-C chemokine receptor type 4
ECM: Extracellular matrix
EGF: Epidermal growth factor
EGFR: Epidermal growth factor receptor
EMT: Epithelial-to-mesenchymal transition
ERK: Extracellular signal-regulated kinase
ESCRT: Endosomal sorting complex required for transport
Fas-L: Fas ligand
HCC: Hepatocellular carcinoma
HCV: Hepatitis C virus
HER2: Human epidermal growth factor receptor 2
HES1: Hes family BHLH transcription factor 1
HES5: Hes family BHLH transcription factor 5
HGF: Hepatocyte growth factor
HSP105: Heat shock protein 105
Hsp70: Heat shock protein 70
Hsp72: Heat shock protein 72
IAP: Inhibitors of apoptosis
IL-2: Inteleukin-2
IL6: Interleukin-6
ILV: Intraluminal vesicle
JAK: Janus kinase
MAPK: Mitogen-activated protein kinase
MDR: Multidrug resistance proteins
MDSC: Myeloid-derived suppressor cells
MEK: MAPK/ERK Kinase
MICA: MHC class 1 related chain ligand A
MIF: Migration inhibitory factor
MLCK: Myosin light-chain kinase
MMP9: Matrix metalloproteinase 9
MRP1: Multidrug resistance-associated protein 1
MSCs: Mesenchymal stem cells
MT-1-MMP: Membrane-type-1 matrix metalloproteinase
MVE: Multivesicular endosome
NK: Natural killer
NKG2D: NKG2-D-activating natural killer receptor
NOTCH2: Neurogenic locus *notch* homolog protein 2
NOTCH3: Neurogenic locus *notch* homolog protein 3
nSMase2: Neutral sphingomyelinase 2
PAD2: Peptidylarginine deiminase 2
PAD4: Peptidylarginine deiminase 4
PAR-2: Protease-activated receptor 2
PCP: Planar cell polarity
PD-1: Programmed cell death receptor 1
PDGF: Platelet-derived growth factor
PDGFRβ: Platelet-derived growth factor receptor-beta
PD-L1: Programmed death ligand 1
PGE2: Prostaglandin E2
P-gp: P-glycoprotein
PI3K: Phosphatidylinositol-3-kinase
PLD: Phospholipase D
PLP: Proteolipid protein
PMEL: Premelanosome protein
PS: Phosphatidylserine
PSAP motif: Proline-serine-alanine-proline motif
PTEN: Phosphatase and tensin homolog
Raf: Raf-1 proto-oncogene, serine/threonine kinase
SDF1: Stromal cell-derived factor 1
SERPINA1: Serpin family A member 1
SERPINF2: Serpin family F member 2
SOCS5: Suppressor of cytokine signaling 5
STAT: Signal transducer and activator of transcription
TF: Tissue factor
TGF-β: Transforming growth factor-beta
TME: Tumor microenvironment
TNF-α: Tumor necrosis factor alpha
TSG101: Tumor susceptibility gene 101
tTG: Tissue transglutaminase
VEGF: Vascular endothelial growth factor
VPS4: Vacuolar protein sorting 4
VTA-1: Vesicle trafficking 1
Wnt4: Wingless-type MMTV integration site family, member 4
WWP2: WW Domain containing E3 ubiquitin protein ligase 2
ZO-1: Zonula occludens protein 1
